# SERTA Domain Containing Protein 1 (SERTAD1) Interacts with Classical Swine Fever Virus Structural Glycoprotein E2, Which Is Involved in Virus Virulence in Swine

**DOI:** 10.3390/v12040421

**Published:** 2020-04-09

**Authors:** Elizabeth A. Vuono, Elizabeth Ramirez-Medina, Paul Azzinaro, Keith A. Berggren, Ayushi Rai, Sarah Pruitt, Ediane Silva, Lauro Velazquez-Salinas, Manuel V. Borca, Douglas P. Gladue

**Affiliations:** 1 Plum Island Animal Disease Center, ARS, USDA, Greenport, NY 11944, USA; elizabeth.vuono@usda.gov (E.A.V.); elizabeth.ramirez@usda.gov (E.R.-M.); Paul.Azzinaro@usda.gov (P.A.); keithab@princeton.edu (K.A.B.); ayushi.rai@usda.gov (A.R.); sarah.pruitt@usda.gov (S.P.); ediane.silva@usda.gov (E.S.); Lauro.velazquez@usda.gov (L.V.-S.); 2 Department of Pathobiology and Population Medicine, Mississippi State University, P.O. Box 6100, Starkville, MS 39762, USA; 3 Department of Pathobiology and Veterinary Science, University of Connecticut, Storrs, CT 06269, USA; 4 Oak Ridge Institute for Science and Education (ORISE), Oak Ridge, TN 37830, USA; 5 Department of Anatomy and Physiology, Kansas State University, Manhattan, KS 66506, USA

**Keywords:** CSFV, CSF, classical swine fever virus, virus-host interactions, SERTAD1

## Abstract

E2 is the major structural glycoprotein of the classical swine fever virus (CSFV). E2 has been shown to be involved in important virus functions such as replication and virulence in swine. Using the yeast two-hybrid system, we previously identified several host proteins specifically interacting with CSFV E2. Here, we analyze the protein interaction of E2 with SERTA domain containing protein 1 (SERTAD1), a factor involved in the stimulation of the transcriptional activities of different host genes. We have confirmed that the interaction between these two proteins occurs in CSFV-infected swine cells by using a proximity ligation assay and confocal microscopy. Amino acid residues in the CSFV E2 protein that are responsible for mediating the interaction with SERTAD1 were mapped by a yeast two-hybrid approach using a randomly mutated E2 library. Using that information, a recombinant CSFV mutant (E2ΔSERTAD1v) that harbors substitutions in those residues mediating the protein-interaction with SERTAD1 was developed and used to study the role of the E2-SERTAD1 interaction in viral replication and virulence in swine. CSFV E2ΔSERTAD1v, when compared to the parental BICv, showed a clearly decreased ability to replicate in the SK6 swine cell line and a more severe replication defect in primary swine macrophage cultures. Importantly, 80% of animals infected with E2ΔSERTAD1v survived infection, remaining clinically normal during the 21-day observational period. This result would indicate that the ability of CSFV E2 to bind host SERTAD1 protein during infection plays a critical role in virus virulence.

## 1. Introduction

Classical swine fever (CSF) is a highly contagious disease of swine with important economic consequences. The disease is caused by classical swine fever virus (CSFV), an enveloped virus with a positive-sense single-stranded RNA genome. The CSFV genome is 12.5 kb, containing a single open reading frame that encodes a 3898-amino-acid polyprotein that yields 11 to 12 final cleavage products (NH2-Npro-C-Erns-E1-E2-p7-NS2-NS3-NS4A-NS4B-NS5A-NS5B-COOH) by the processing of the polyprotein by viral and cellular proteases [1].

CSF is found in Central and South America, Europe, Asia and parts of Africa. Recently, in 2018, CSF outbreaks were reported in Japan after it had been free of the disease for twenty-six years [2]. Despite vaccine control strategies using the C-strain, a vaccine developed by serial passage in rabbits that led to its attenuation and that has been used as an effective vaccine against CSFV, the disease has continued to be endemic in several countries. Recently, in China, the emergence of subgenotype 2.1d occurred, causing mild pathological and histological lesions in C-strain vaccinated swine, suggesting that although no clinical symptoms are observed, the C-strain would not provide pathological and virological protection against the emerging subgenotypes [3]. A similar occurrence has also been observed in Cuba, where selective pressure has resulted in escape mutants from vaccination with the C-strain. These CSFV escape mutants are defined to a small cluster in genotype 1.2 that has mutations in the B/C domains of the structural protein E2. This new cluster of genotype 1.2 in CSFV results in a mild disease in C-strain vaccinated animals [4]. Therefore, research focused on the better understanding of virus–host protein-protein interactions in the natural host could potentially lead to the continued development of novel tools to prevent infection or disease. 

There are four structural proteins in CSFV, the core protein and three envelope glycoproteins, E^rns^, E1 and E2, which compose the CSFV particle. Mutations in the structure proteins have been linked to CSFV being able to escape vaccination strategies [3,4]. Understanding the molecular functions of these proteins, in particular, of specific residues that are involved in the processes of virus replication and virulence, has begun to be studied in the last few years [5,6,7,8,9,10,11] but is limited to only a few reports involving specific residues of these proteins. 

The identification of cell proteins specifically interacting with virus proteins during infection is a relatively new field of CSFV research. The CSFV core protein has been shown to interact with IQGAP1 (IQ motif containing GTPase activating protein 1), UBC9 (Ubiquitin Conjugating enzyme 9), SUMO1 (small ubiquitin-related modifier 1) and HB (Hemoglobin subunit beta) [12,13,14,15]. E^rns^ has been demonstrated to interact with the laminin receptor [16]. In addition, E2 has been shown to interact with several host proteins including cellular actin [17], thioredoxin [18], annexin 2 [19], mitogen-activated protein kinase 2 [20], protein phosphatase 1 catalytic subunit beta [21] and dynactin 6 [22]. These host–virus protein-protein interactions have been shown to play a role in regulating the virus replication cycle, and in some cases, these protein interactions are involved in virus virulence [12,13,14,22]. 

Previously, we have identified, using a yeast two-hybrid approach (YTH), several swine host proteins interacting with CSFV E2 [23]. One of these proteins was SERTA domain containing protein 1 (SERTAD1), a factor involved in the modulation of the transcriptional activities of different host genes. Here, we expand our preliminary report by analyzing the protein-protein interaction between E2 and SERTAD1. The interaction occurring between these two proteins was demonstrated to occur in CSFV-infected swine cells by the proximity ligation assay and confocal microscopy. The E2 amino acid residues critical for interaction with the SERTAD1 protein were mapped by a yeast two-hybrid assay, and based on that information, a recombinant CSFV mutant (E2ΔSERTAD1v) was developed by reverse genetics containing residue substitutions disrupting the E2-ΔSERTAD1 interaction. CSFV E2ΔSERTAD1v showed a decreased replication ability when compared with the parental virus in the swine cell line SK6 and was still less efficiently replicated in primary swine macrophages. Interestingly, most animals infected with CSFV E2ΔSERTAD1v survived infection, indicating that the amino acid residues of E2 identified to bind host SERTAD1 protein may play an important role in CSFV virulence in swine.

## 2. Materials and Methods

### 2.1. Viruses and Cells

Swine kidney cells (SK6), free of BVDV, were cultured in Dulbecco’s Minimal Essential Media (DMEM) (Gibco, Grand Island, NY, USA) with 10% fetal calf serum (FCS) (Atlas Biologicals, Fort Collins, CO, USA). The CSFV Brescia strain was propagated in SK6 cells and used for the construction of an infectious cDNA clone (IC) [5]. BICv is the virus derived from the IC [5]. Growth kinetics were assessed for both SK6 cells and primary swine macrophage cell cultures prepared as described [5]. The titration of CSFV from clinical samples was performed using SK6 cells in 96-well plates (Costar, Cambridge, MA, USA). Viral infectivity was detected after 4 days in culture by an immunoperoxidase assay using the CSFV monoclonal antibody WH303 [24] and the Vectastain ABC kit (Vector Laboratories, Burlingame, CA, USA). Titers were calculated as previously described [25] and expressed as TCID_50_/mL As performed, the test sensitivity was ≥1.8 TCID_50_/mL Plaque assays were performed using SK6 cells in 6-well plates (Costar). SK6 monolayers were infected, overlaid with 0.5% agarose and incubated at 37 °C for 3 days. Plates were fixed with 50% (vol/vol) ethanol-acetone and stained by immunohistochemistry with the monoclonal antibody WH303 [24].

### 2.2. Proximity Ligation Assay

The Proximity Ligation Assay (PLA) was performed exactly as described elsewhere [21]. Briefly, the assay was run in triplicate following the guidelines from the Duolink-PLA kit (DUO92101). SK6 cells were plated onto 12 mm round coverslips (Thomas Scientific, Swedesboro, NJ, USA) in a 24-well plate (Corning, Corning, NY, USA) at a density of 25,000 cells/well. Next day, cells were infected (MOI = 10) with BICv, and 24 h later, the cells were fixed with 4% formaldehyde w/v in PBS at room temperature for 20 min, followed by soaking with permeabilization buffer (0.3% Triton-X-100 in PBS) for 10 min. Fixed cells were then blocked with the Duolink blocking buffer for 30 min at 37 °C, followed by incubation with the primary antibodies anti-E2 WH303 [24] and anti-SERTAD1 (Abcam cat# ab65446, Cambridge, UK) at 4 °C for 1 h. Cells were then washed twice with the Duolink (Sigma-Aldrich) wash buffer A and incubated with the PLUS and MINUS PLA probes for 1 h at 37 °C, followed by 2 washes with the Duolink wash buffer A, and a 30 min incubation at 37 °C with the Duolink Ligase in ligation buffer. Fixed cells were then washed twice with the Duolink wash buffer A followed by incubation with Duolink polymerase in the Amplification buffer at 37 °C for 100 min. The fixed cells were then washed twice with the Duolink wash buffer B and mounted with the Duolink PLA Mounting Medium with DAPI.

### 2.3. Yeast Two-Hybrid Screening for the Disruption of SERTAD1’s Binding to E2

Plasmids E2-BD and SERTAD1-AD were previously identified or constructed [23]. E2-BD was randomly mutated using a mutagenic PCR approach to give an average of 5 nucleotide substitutions across the E2 open reading frame (this random mutant library was constructed by Epoch Bioscience (Bothell, WA)). The random mutants library was then co-transformed into the yeast strain AH109 along with SERTAD1-AD with a transformation rate of at least 1 × 10^6^ individual colonies, representing full coverage of the E2 mutagenic library. Screening for the disruption of the E2 binding domains for SERTAD1-AD was performed as previously described [14]. The sequencing of the disruption of binding mutants in E2 often revealed stop codons or out of frame mutations, thus explaining why the loss of E2-SERTAD1 protein-protein interactions occurred; these plasmids were discarded. When individual amino acids were mutated, the E2 mutant plasmids were tested by co-transformation with SERTAD1 and HPRT1-AD (hypoxanthine phosphoribosyl transferase 1), a previously identified [23] positive E2 protein interactor, or PGADT7 (negative control) and selected on minimal media lacking amino acids leucine and tryptophan (SD-TL) plates. Then, individual colonies were grown overnight in SD-TL liquid media at 30 °C and spot plated on both SD-TL and SD-ALTH (minimal media lacking amino acids leucine, tryptophan, adenine and histidine) as selective media to assess the ability of individual E2 mutants to bind both positive and negative controls (HPRT1-AD and PGADT7, respectively) and to confirm loss of binding to SERTAD1. This second selection was performed to discard any mutant E2 proteins that lost the ability to bind SERTAD1 because of a gross structural change in E2, rather than through the loss of a residue critical for binding SERTAD1.

### 2.4. Co-Localization

SK6 cells were plated in 24-well tissue culture dishes containing 12 mm round coverslips at a density of 2.5 × 10^4^ cells per/well and infected with BICv at an MOI of 0.1 for 24 h or mock infected. Twenty-four hours later, the cells were fixed on the coverslips with 4% paraformaldehyde (EMS, Hatfield, PA) for 15 min. The paraformaldehyde was removed and the tissue culture dishes were washed three times with PBS, and then the cells were permeabilized with 0.5% Triton X-100 for 5 min at room temperature, followed by incubation in blocking buffer (phosphate-buffered saline [PBS], 5% normal goat serum, 2% bovine serum albumin, 10 mM glycine, 0.01% Thimersal) for 1 h at room temperature. The fixed cells were then incubated with the primary antibodies for E2 and SERTAD1 overnight at 4 °C. After being washed three times with PBS, the cells were incubated with the appropriate secondary antibodies, goat anti-rabbit immunoglobulin G (IgG) (1/400; Alexa Fluor 594, Molecular Probe) and goat anti-mouse immunoglobulin G (IgG Alexa Fluor 488; Molecular Probes), for 1 h at room temperature. After this incubation, the coverslips were washed three times with PBS, and mounted with ProLong Gold Antifade Mountant (Life Technologies) containing D and then left for 24 h to cure. The samples were examined using a Zeiss LSM 710 confocal microscope. The captured images were adjusted for contrast and brightness using the Zeiss Zen Blue software.

### 2.5. Construction of the CSFV E2ΔSERTAD1v Mutant

A full-length Infectious Clone of the virulent CSFV Brescia strain (pBIC) [5] was used as a template to introduce E2 amino acid substitutions disrupting E2-SERTAD1 protein-protein interactions. These amino acid substitutions were identical to those mapped by the reverse yeast two-hybrid methodology. Residue substitutions T149A, Y325H and H335R were introduced into the native E2 sequence in the designed pBICΔSERTAD1 construct. The pBICΔSERTAD1 plasmid was obtained by DNA synthesis (Epoch Life Sciences, Sugar Land, TX, USA). 

The CSFV pBICΔSERTAD1 full-length genomic clone was linearized with *SrfI* and in vitro transcribed using the T7 MEGAscript system (Ambion, Austin, TX). RNA was precipitated with LiCl and transfected into SK6 cells by electroporation at 500 volts, 720 ohms and 100 watts with a BTX 630 electroporator (BTX, San Diego, CA, USA). Cells were seeded in 6-well plates and incubated for 4 days at 37 °C and 5% CO_2_. Virus was detected by immunoperoxidase staining as described above, and stocks of rescued viruses were stored at ≤−70 °C. The full-length genome of the in vitro rescued CSFV E2ΔSERTAD1 virus (E2ΔSERTAD1v) was completely sequenced by NGS.

### 2.6. Ethics Statement

Animal experiments were performed under biosafety level 3AG conditions in the animal facilities at the Plum Island Animal Disease Center (PIADC). All experimental procedures were carried out in compliance with the Animal Welfare Act (AWA); the 2011 Guide for Care and Use of Laboratory Animals; the 2002 PHS Policy for the Humane Care and Use of Laboratory Animals; and U.S. Government Principles for Utilization and Care of Vertebrate Animals Used in Testing, Research and Training (IRAC 1985); as well as specific animal protocols reviewed and approved by the PIADC Institutional Animal Care and Use Committee of the US Departments of Agriculture and Homeland Security (protocol number 171.05-18-R Classical swine fever virus (CSFV): evaluation of virulence of wild type and genetically modified viruses; approved on 2 October 2018). 

### 2.7. Animal Infections

The E2ΔSERTAD1v mutant was evaluated for its virulence phenotype in swine relative to the virulent Brescia strain. The swine used in all animal studies were 10 to 12 week old, forty-pound commercial breed pigs. Five animals were inoculated intranasally with 10^5^ TCID_50_ of either E2ΔSERTAD1v or wild-type parental virus (BICv). Clinical signs (anorexia, depression, purple skin discoloration, staggering gait, diarrhea and cough) and changes in body temperature were recorded daily throughout the 21-day experiment. Total and differential white blood cell, lymphocyte and platelet counts were obtained using a Hemavet HV950FS (Drew Scientific Inc., Miami Lakes, FL, USA).

## 3. Results

### 3.1. CSFV E2 and Swine Host Protein SERTAD1 Interact in CSFV-Infected Cells

Two independent methodologies were used to confirm the yeast two-hybrid results showing that E2 and SERTAD1 protein-protein interactions take place during CSFV cell infection.

**Figure 1:**
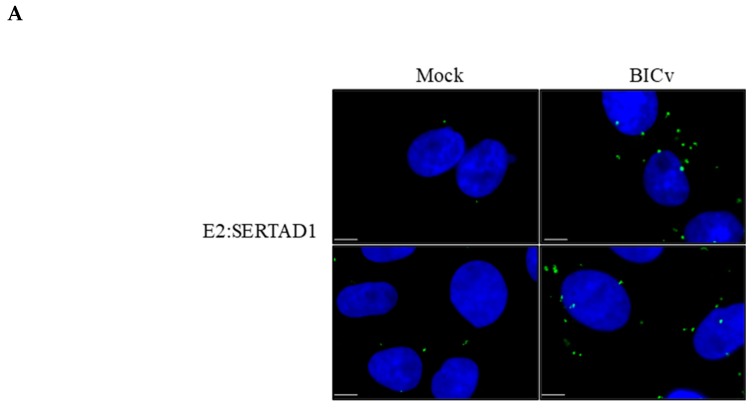

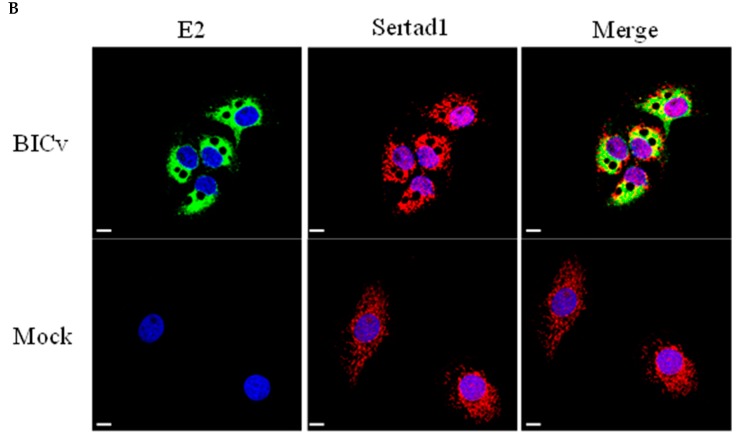
The interaction between classical swine fever virus (CSFV) E2 and SERTAD1 in CSFV-infected SK6 cells demonstrated by (**A**) the proximity ligation assay (PLA) (the top and bottom panels are images showing different fields within the same treatment) and (**B**) confocal microscopy in SK6 cells that were either mock-infected or infected for 24 h with CSFV BICv. The size bars are 10 µm.

First, a proximity ligation assay (PLA) [26], which allows the identification of transient protein-protein interactions, was employed to detect E2-SERTAD1 interactions. SK6 cells were infected (MOI = 10) with BICv. Samples were harvested at 24 h post-infection (hpi) and processed as described in Materials and Methods. The results of the PLA confirmed that E2 and SERTAD1 interact in SK6 cell cultures infected with CSFV. This interaction appears as a distinct punctate location throughout the cell cytoplasm (Figure 1A). These results indicated that in CSFV-infected cells, E2 specifically interacts with SERTAD1, confirming the previous yeast two-hybrid findings that suggested that E2-SERTAD1 protein-protein interactions occur during the viral infection of cell cultures.

To further confirm the yeast two-hybrid and PLA results, we performed co-localization studies between SERTAD1 and E2 in SK6 cells infected with CSFV. The co-localization of E2 and SERTAD1 was detected as punctate structures homogeneously distributed in the cytoplasm of the infected cells (Figure 1b). The signal for PLA occurs only when the two interacting proteins are close enough, allowing the complementary oligonucleotides that are attached to the secondary antibodies to ligate together. Consequently, signal location does not necessary indicate all intracellular sites where either of the proteins may be expressed or co-localize. Therefore, using two different methodologies, it is shown that the E2 and SERTAD1 proteins interact in CSFV-infected cells.

### 3.2. Identification of CSFV E2 Residues Critical for SERTAD1 Interaction

**Figure 2:**
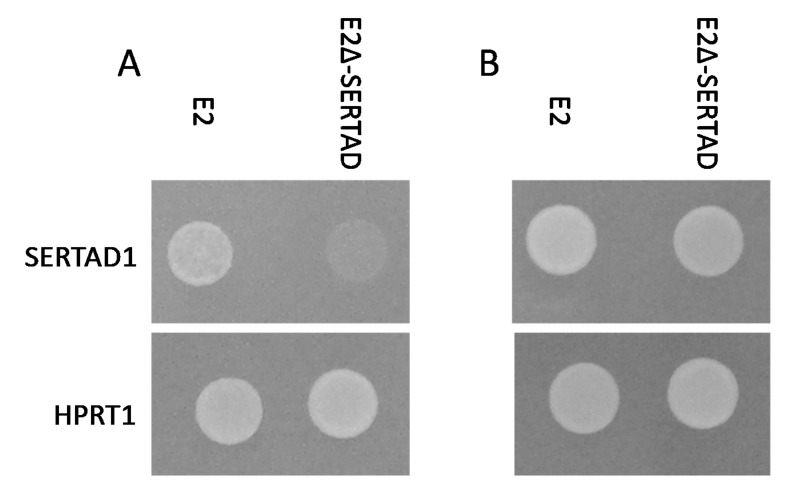
The yeast two-hybrid protein-protein interaction of CSFV protein E2 or E2-∆SERTAD (T149A, Y325h and H335R) coupled to the Gal4 binding domain with SERTAD1 or HPRT coupled to the Gal4 activation domain. Spots of strains (10 uL), expressing the indicated constructs were spotted on (**A**) selective media SD-Ade/His/Leu/Trp or (**B**) non-selective media SD-Leu/Trp.

The development of mutant viruses containing residue substitutions disrupting interactions between a virus protein and its host cell partner constitutes a useful tool to analyze the potential role of a particular E2-host protein interaction in several virus functions [12,13,14,27]. We have previously reported that the E2-SERTAD1 interaction appears to be conformation-dependent since alanine scan mutagenesis failed to map E2 residues critical for mediating interaction [23], an approach successfully used by us to map residues within the foot and mouth disease virus (FMDV) and CSFV proteins interacting with host protein ligands [14,27,28,29,30]. E2 amino acids mediating the interaction with SERTAD1 were mapped using the yeast two-hybrid methodology as described in our previous study [22]. The methodology evaluates the ability of SERTAD1 to interact with a library of randomly mutated versions of E2, harboring an average of five amino acid residues that are randomly substituted. Mutated forms of E2 lacking reactivity with SERTAD1 were also tested for their ability to bind protein HPRT1, another host protein that also specifically binds E2, to rule out the possibility that the random E2 residue substitutions caused gross conformational changes in E2 that could disrupt the overall structure of E2, resulting in a nonspecific loss of protein binding (Figure 2). Only one E2 mutant was able to fulfill all of the criteria while still losing the ability to bind SERTAD1. This mutant configuration of E2, harboring substitutions at positions T149A, Y325H and H335R, was used to further study the role of SERTAD1’s binding to E2 in CSFV replication and virulence. 

### 3.3. Development of the CSFV E2ΔSERTAD1v Mutant 

To functionally assess the potential role of the E2-SERTAD1 interaction, a recombinant mutant virus based on the virulent strain Brescia was designed using reverse genetics. The amino acid residues of E2 identified as necessary for the E2-SERTAD1 interaction using the yeast two-hybrid approach were mutated (residue changes at E2 positions T149A, Y325H and H335R) and then incorporated into a full-length cDNA copy of the CSFV strain Brescia (pBIC) [5], producing the pE2ΔSERTAD1 construct. pE2ΔSERTAD1-derived infectious RNA was obtained by in vitro transcription and used to transfect SK6 cells. CSFVs BICv and E2ΔSERTAD1v were rescued from transfected cells at 4 dpi (days post-infection). The nucleotide sequences of viable rescued virus genomes were identical to those of parental DNA plasmids, confirming that only mutations at the predicted mutated sites were present in rescued viruses.

### 3.4. Replication of E2ΔSERTAD1v Mutants In Vitro

**Figure 3:**
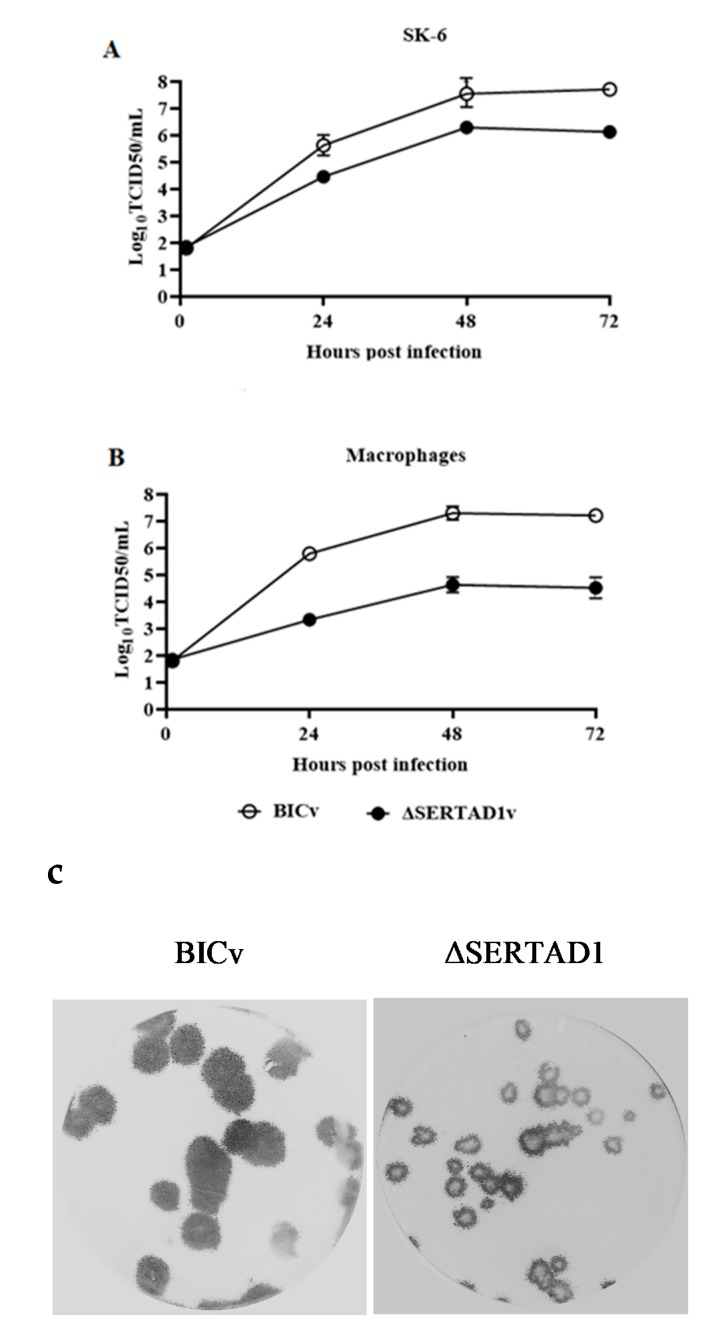
The in vitro growth characteristics of CSFV E2ΔSERTAD1v. The multistep growth curves of E2ΔSERTAD1v and BICv in (**A**) SK6 cells and (**B**) swine macrophage cell cultures. Cell cultures were infected (MOI of 0.01) with CSFV BICv (empty symbols) or E2ΔSERTAD1v (filled symbols). At the indicated times post-infection, samples were collected and titrated for virus yield. Data are represented as the means and standard deviation of three independent experiments. (**C**) Plaque formation of CSFV E2ΔSERTAD1v and BICv on SK6 cell cultures. Cell cultures were infected with either virus and stained 4 days later as described in Materials and Methods.

The in vitro growth characteristics of mutant virus E2ΔSERTAD1v were compared with those of parental BICv (the pBIC-derived virus) in a multiple-step growth curve using both SK6 and primary swine macrophage cultures. Cell cultures were infected at a multiplicity of infection (MOI) of 0.01 TCID_50_ per cell. Virus was adsorbed for 1 h (time zero), and samples were collected daily until 72 h post-infection (hpi). As expected, BICv displayed similar growth kinetics in SK6 cells and swine macrophages. Conversely, E2ΔSERTAD1v exhibited a small reduction in virus replication in SK6 cells compared with in parental BICv but presented a significant decrease in virus replication in swine macrophages, showing a reduction in virus yield of approximately 100-fold compared to that in BICv-infected macrophages (Figure 3A,B).

In accordance with its decreased growth ability, the plaque size of E2ΔSERTAD1v in SK6 cell cultures was reduced by approximately 50% relative to the BICv plaque size (Figure 3C). It is clear from these experiments that E2 residue substitutions that disrupt E2-SERTAD1 interactions in yeast two-hybrid appears to affect the ability of CSFV to replicate in cell cultures, particularly in primary swine macrophages. 

### 3.5. Evaluation of E2ΔSERTAD1v in Virulence in Swine

**Figure 4:**
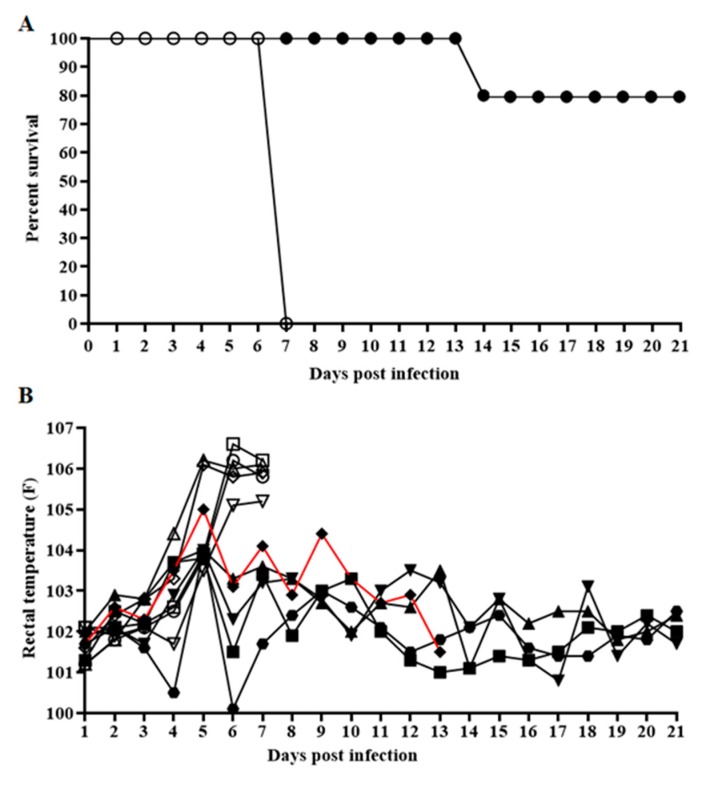
The evolution of mortality (**A**) and body temperature (**B**) in animals intransally (IN) infected with 10^5^ TCID_50_ of either E2ΔSERTAD1v (filled symbols) or parental BICv (open symbols). Animals were monitored for an observational period of 21 days post infection. In red is the evolution of the rectal temperatures of the only E2ΔSERTAD1v-infected animal euthanized at 14 days post infection (dpi).

To assess the effect of the potential disruption of the E2-SERTAD1 interaction on CSFV virulence, mutant E2ΔSERTAD1v or parental BICv was intranasally (IN) inoculated into naïve swine (10^5^ TCID_50_), which were monitored for the appearance of clinical signs associated with the disease for up to 21 days. As expected, animals infected with BICv displayed a characteristic disease with a rise in body temperature by day 5 post infection (pi), followed by the appearance of classic clinical signs associated with CSF (Table 1 and Figure 4). All pigs needed to be humanely euthanized at around day 7 pi due to the severity of the clinical signs. Conversely, animals inoculated with mutant E2ΔSERTAD1v showed a milder disease. Four animals survived during the observational period of 21 days, remaining clinically normal with only a transient rise in body temperature by day 5 pi. The fifth animal presented a milder disease compared to those inoculated with BICv that progressed until day 14, when it was euthanized due to the severity of the disease (Table 1 and Figure 4).

**Figure 5:**
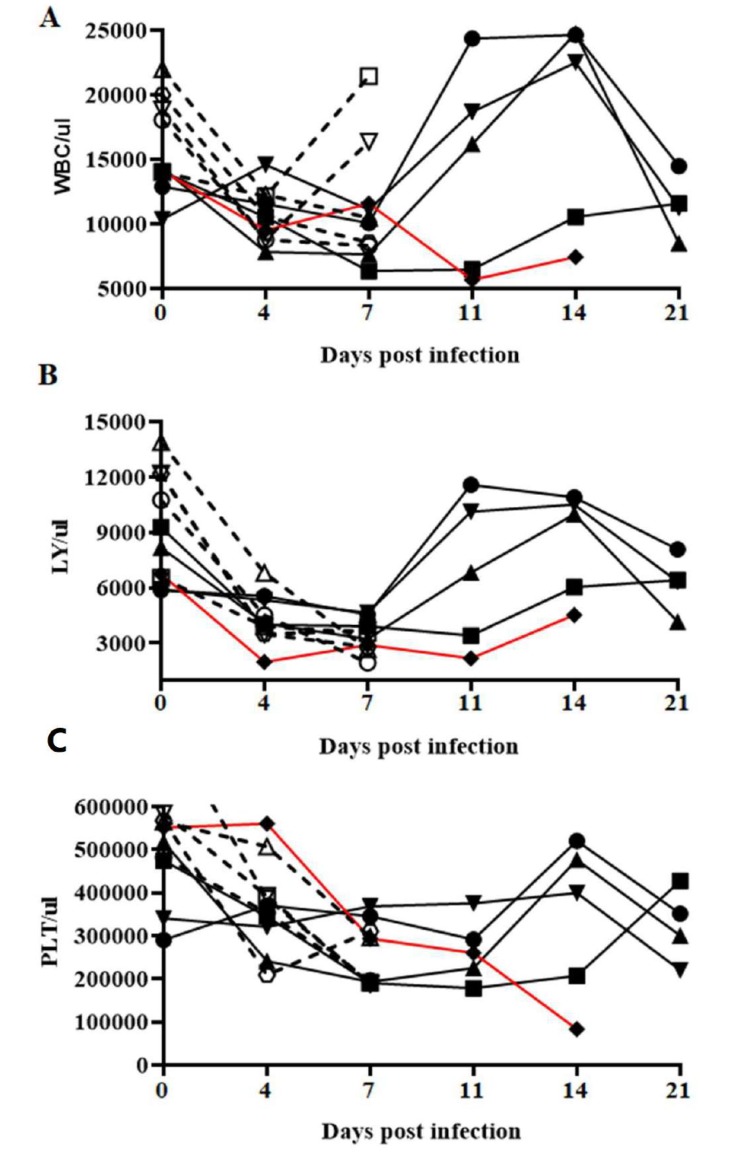
The concentrations of circulating white blood cells (**A**), lymphocytes (**B**) and platelets (**C**) in animals IN infected with 10^5^ TCID_50_ of either E2ΔSERTAD1v (filled symbols) or parental BICv (open symbols). Animals were monitored for an observational period of 21 days post infection. In red is the evolution of the cell values of the only E2ΔSERTAD1v-infected animal euthanized at 14 dpi.

Circulating counts of white blood cells (WBCs), lymphocytes and platelets were analyzed, as they are known indicators of CSF severity (Figure 5). In BICv-infected animals, circulating WBC values dropped drastically by 4 dpi and either remained low or slightly increased until the animals were euthanized by 7 dpi. Animals infected with E2ΔSERTAD1v also showed a drastic drop in WBC values by 4 dpi, followed by a clear recovery by 11–14 dpi and a new drop by the end of the experimental period, with the exception of the animal euthanized at 14 dpi, in which counts remained low. Blood lymphocyte and platelets counts in BICv-infected animals also dropped by day 4 and continued to decrease until animals were euthanized at 7 dpi. A clear decrease was observed in blood lymphocyte and circulating platelet counts by days 4 and 7 post infection, respectively, in E2ΔSERTAD1v-infected animals, with this transient drop followed by a return to near-normal values by 11–14 dpi, followed by a slow decrease in numbers at the end of the observational period. As happened with circulating white cell counts, blood lymphocytes and circulating platelet values in the E2ΔSERTAD1v-infected animal euthanized at 14 dpi remained low until death. In summary, infection with E2ΔSERTAD1v provoked a gradual decrease in hematological values that occasionally returned to baseline values during the 21-day observational period. This indicates that E2ΔSERTAD1v-infected animals still retain some degree of virulence even though the animals do not present clinical signs of the disease.

**Figure 6:**
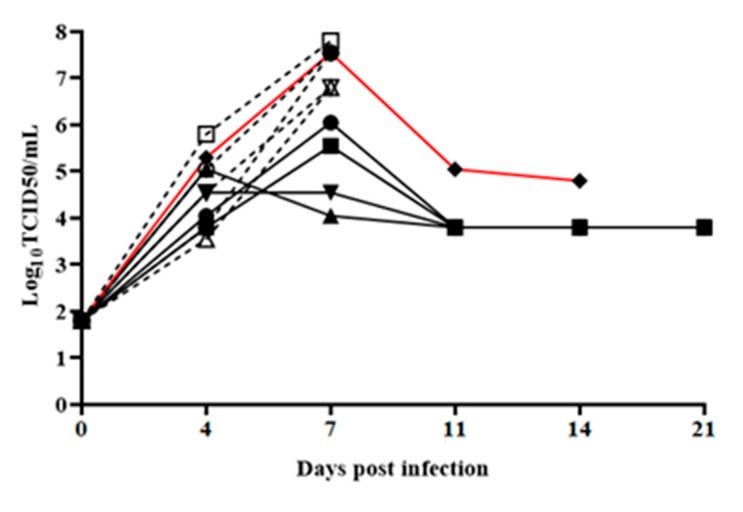
The virus titers in blood samples obtained from in animals IN infected with 10^5^ TCID_50_ of either E2ΔSERTAD1v (filled symbols) or parental BICv (open symbols). Values are expressed as log_10_ TCID_50_/mL. Sensitivity of virus detection: ≥10^1.8^ TCID_50_/mL. In red is the evolution of viremia of the only E2ΔSERTAD1v-infected animal euthanized at 14 dpi.

Virus replication, detected as viremia, closely followed the presentation of clinical signs. In BICv-inoculated animals, titers were clearly detectable (ranging between 10^4^ to 10^5^ TCID_50_/mL) by 4 dpi, noticeably increasing to high values (ranging between 10^6.5^ to 10^8^ TCID_50_/mL) until the animals were euthanized by 7–8 dpi. Viremic titers in animals inoculated with E2ΔSERTAD1v were similar to those in BICv-inoculated animals by 4 dpi (averaging 10^4.5^ TCID_50_/mL, SD 0.32), remained relatively low by 7 dpi, with titers approximately 1000-fold lower than in BICv-infected animals. Viremias remained stable but detectable in CSFV E2ΔSERTAD1v-infected animals until the end of the experimental period (Figure 6). Interestingly, viremia values from the E2ΔSERTAD1v-infected animal that was euthanized on day 14 were not drastically different than the titers in the surviving E2ΔSERTAD1v-infected animals. 

Based on the appearance of clinical signs associated with CSF, hematological values and the levels of virus replication in animals infected with CSFV E2ΔSERTAD1v, a correlation exists between the disruption of the E2-SERTAD1 interaction and a decrease in virus virulence during the infection in domestic swine. 

## 4. Discussion

Although there is some information available regarding the molecular mechanisms developed by CSFV to increase virus replication and to manipulate the host immune response, the role of virus–host protein-protein interactions in the outcome of the disease is still not well understood. As a result of the interaction between virus and host protein(s), the virus may modulate the host cell response, permitting the virus to manipulate host metabolic pathways to facilitate its replication. Previously, we have studied the interaction of host proteins with several CSFV proteins. We have shown that the structural CSFV core protein interacts with host SUMO1, IQGAP1, UBC9 and OS9 [12,13,14,15]; shown that p7, a viroporin, interacts with CAMLG [28]; and more recently, described the interaction of the major structural glycoprotein E2 with PPP1CB and DCNT6 [21,22]. Importantly, besides affecting virus replication, some of these host–virus protein interactions play a role in determining virus virulence in swine [12,13,14,22]. Understanding the host protein involvement for CSFV is critical to further understand the mechanisms involved during viral infection of the host. We report here the characterization of the interaction between the CSFV E2 protein and SERTAD1, a regulator of cell transcription. 

The SERTAD1 gene encodes a 236 amino acid protein harboring five potential functional domains [31,32,33,34]: (i) an area with homology to the Cyclin A binding sequence (between residues 1 and 30) (although whether SERTAD1 actually interacts with Cyclin A has not been experimentally determined); (ii) a second domain (between residues 43 and 82) represented by a novel heptad hydrophobic repeat that has been identified in multiple proteins from insects to humans (although its exact biological activities remain unclear); (iii) a cyclin-dependent kinase 4 (CDK4) binding domain (between residues 30 and 160), which is responsible for the direct interaction between CDK4 and SERTAD1 [34]; (iv and v) two partially overlapping transactivation domains: the PHD-bromodomain-interacting domain (in residues 161–178), which interacts with the bromodomain and/or PHD zinc finger-containing general transcriptional co-activators (such as p300/CBP), and an acidic region (residues 167–220), which has intrinsic transactivation activity [31,32,33]. In addition to regulating gene expression, SERTAD1 directly interacts with CDK4 to antagonize the activity of the cdk inhibitor p16INK4, thereby promoting cell proliferation [34,35]. It is known that SERTAD1 forms a complex with transcription factors (such as E2F1) to co-activate the transcription of target genes [32,33]. To our knowledge, there have not been reports involving SERTAD1 as a binding partner of any viral protein nor of the participation of SERTAD1 in the process of the modulation of virulence in any viral infection.

The results reported here characterize, for the first time, the cellular protein SERTAD1 as an interaction partner for CSFV protein E2 in the infected cell. These results indicate that the E2-SERTAD1 protein-protein interaction is important for CSFV replication in vitro. Mutations that disrupt the E2-SERTAD1 protein-protein interaction in YTH provokes a significant change of the E2ΔSERTAD1v phenotype affecting virus replication in vitro and virus virulence in vivo. The mutation of these specific residues appears critical for virus virulence during CSFV infection in the natural host, the domestic swine. This presents new possibilities to explore CSFV pathogenesis and the virus requirements of virus-host interactions necessary to produce disease. 

Understanding the role of specific residues of important CSFV structural proteins, such as E2, is necessary for developing more effective next generation vaccines. A more complete understanding of the host factors interacting with E2 at the amino acid level is an important step to gain a better understanding of the impact of potential mutations that occur between different emerging strains of CSFV, potentially helping to explain the differences observed with regard to viral virulence and pathogenesis. 

## Figures and Tables

**Table 1:** Swine survival and fever responses in animals infected with mutant E2ΔSERTAD1v compared with those infected with parental BICv.

			Fever		
Treatment ^1^	No. of Survivors/Total	Mean Time to Death (Days ± SD)	No. of Days to Onset (Days ± SD)	Duration No. of Days (Days ± SD)	Maximum Daily Temp (°F ± SD)
BICv	0/5	7 (0)	5.2 (0.84)	1.8 (0.84)	105.82 (0.44)
CSFV E2ΔSERTAD1v	4/5	14 ^2^	5 ^3^	9 ^2^	104.16 (0.47) ^3^

^1^ All animals were IN inoculated with 10^5^ TCID_50_ of the indicated virus. Animals were observed for 21 days after inoculation. ^2^ Data are based on the only animal in the group that needed to be humanely euthanized. ^3^ All animals in the group presented a transient rise in body temperature by day 5 post infection.
